# Complete genome of *Streptomyces anulatus* DEF39, an endophytic biocontrol agent against *Fusarium graminearum*

**DOI:** 10.1186/s12863-026-01474-y

**Published:** 2026-08-04

**Authors:** Irene Valenti, Andrea Motta, Marco Saracchi, Andrea Kunova, Cristina Pizzatti, Paolo Cortesi, Matias Pasquali, Daniela Bulgari

**Affiliations:** Department of Food, Environmental and Nutritional Sciences, University of Milan, Milan, Italy

**Keywords:** Actinomycetes, Phylogeny, Metabolic pathways, Secondary metabolites, Plant growth promotion traits

## Abstract

**Objectives:**

Phylogenetic and functional genomic analyses were carried out to help explaining the endophytic lifestyle and the biocontrol activity of *Streptomyces* sp. strain DEF39, able to reduce *Fusarium graminearum* infection as well as deoxynivalenol production in wheat plants. Specifically, in this work, genes and biosynthetic pathways linked to secondary metabolite production, biocontrol activity, and plant interactions were identified. This approach supports the experimentally validated capability of DEF39 to protect plants from fungal diseases, highlighting the potential of genomics studies.

**Data description:**

*Streptomyces* sp. DEF39 was isolated as an endophyte from *Secale cereale* in Italy and has been shown to colonize wheat (*Triticum* spp.) and act as a biocontrol agent of Fusarium head blight. The complete genome (9.12 Mb) (GCA_978019405.1), assembled through a hybrid strategy based on long and short accurate reads combination, is composed of a linear chromosome of 8,887,323 bp with a GC content of 71.59% and a linear plasmid of 233,172 bp with a GC content of 69.27%. Phylogenetic analyses based on the whole genome demonstrated that DEF39 belongs to *Streptomyces anulatus* species (ANI and dDDH value of 99.13% and 92.8%, respectively with the reference genome GCA_001434355.1). Functional annotation revealed 9,556 genes (CDS). Respectively, antiSMASH and PLaBAse bioinformatic tools predicted 15 putative secondary metabolite biosynthetic gene clusters, as well as 5,203 genes putatively associated with plant growth promotion.

## Objective

*Streptomyces* is a wide genus of Gram-positive bacteria belonging to the *Actinobacteria* phylum [1]. *Streptomyces* spp. are soil dwellers and plant symbionts able to produce a plethora of specialized metabolites [2, 3]. Their prolific production of secondary metabolites has made them crucial for natural product research, responsible for most antimicrobial compounds isolated to date [4]. Many bioactive compounds from *Streptomyces* spp. have found applications in medicine as antitumor, antiviral, antifungal molecules, and in agriculture [5–7]. Moreover, different *Streptomyces* spp. have been evaluated as plant growth-promoting bacteria (PGPB), both with biostimulation effects [8] and activities against pathogens [9, 10], found in different plant species.

In recent years, innovative bioinformatic tools based on whole genomes and large curated databases have been replacing traditional taxonomic techniques, providing genome-based taxonomy by employing whole-genome data and phylogenomic methods. Genome sequencing and annotation have greatly enhanced the analysis of secondary metabolite biosynthetic gene clusters (smBGCs) [11]. This has led to the discovery of microorganisms producing unknown bioactive products and the characterization of their biosynthetic pathways. A hybrid sequencing approach, by combining short and long-read technologies, warrants high-quality genomes essential for effective genome mining.

*Streptomyces* sp. DEF39 was previously characterized for its biocontrol activity on Fusarium Head Blight (FHB), showing an endophytic behavior in wheat [12–14]. In vitro and in vivo experiments demonstrated DEF39 ability to suppress deoxynivalenol (DON) production by *F. graminearum* without affecting pathogen growth, as well as its ability to colonize the plant when wheat seeds are treated with DEF39 spores [15].

This work aims to provide a high-quality annotated genome of this strain, including metabolic pathways and secondary metabolite production potential. We also identified genes that might modulate plant fitness and might explain the endophytic behavior of DEF39. 

## Data description

*Streptomyces* sp. DEF39 was isolated as an endophyte from *Secale cereale* in Italy (45.474821, 9.227084). For high-quality DNA extraction, a spore suspension (2.5 × 10^6^ spores) was inoculated in 50 mL of Czapek-Dox broth (35 g/L Czapek Dox broth; 2 g/L yeast extract) and incubated at 24 °C for 14 days at 120 rpm. DNA was extracted using DNeasy UltraClean Microbial Qiagen Kit and quantified using the Qubit fluorometer. Genomic DNA was sequenced using both Illumina and Oxford Nanopore Technologies (ONT) platforms, integrating short and long reads with a coverage of 75x and 87.6x, respectively, for a total sequencing coverage of 163x (Table 1, Data set 1). Illumina sequencing was performed by Eurofins Genomics using Illumina Miseq paired reads method, while the ONT sequencing was performed by the MinION system Mk1B (R9.4.1 flow-cell) following the pipeline of the ligation sequencing kit (SQK-LSK109).

Quality assessment of raw ONT (0.3 Mbp; N50 7953 bp) and Illumina reads (2.76 Mbp per dataset) was carried out using NanoPlot 1.28.2 [16] and FastQC [17], respectively. The DEF39 genome was assembled through a hybrid strategy. Long reads were assembled using Flye 2.9 [18] on Galaxy platform. The assembly (two contigs, N50 = 8.9 Mb) was first polished via neural networks using Medaka consensus pipeline 1.4.4 [19], which improved the genome completeness, increasing the number of complete BUSCO genes (BUSCOs) from 761 to 999 (streptomycetales_odb10 lineage, 1,579 BUSCOs). A second polishing step was performed by mapping Illumina short reads (75× coverage) onto the Medaka-polished assembly using Minimap2 and extracting the consensus with Pilon, further increasing BUSCOs to > 1,500. Short-read contigs were generated using Shovill (N50 = 0.85 Mb, 1,571 complete BUSCOs) while hybrid assemblies (i.e., starting from both short and long reads) were produced with Unicycler 5.0.2 [20] (seven contigs, 1,571 complete BUSCOs). To maximize both contiguity and base-level accuracy, Unicycler contigs were mapped against the Flye-Medaka contigs in Geneious Prime 2021 via Minimap2 alignment. At positions of disagreement, the Unicycler base call (supported by short reads) was preferentially adopted. Long and short reads were then remapped to close gaps, correct indels, and verify critical regions, including terminal regions and contig junctions. Regions with no short-read coverage but covered by long reads were retained. After these steps, the final DEF39 genome of 9.12 Mb (GCA_978019405.1, Table 1, Data set 2), was obtained with a completeness of 99.7% and 99.84% respectively by BUSCO [21] and CheckM v1.2.3 [22]. The genome is composed of a linear chromosome of 8,887,323 bp with a GC content of 71.59% and a linear plasmid of 233,172 bp with a GC content of 69.27% (Table 1, Data File 1). The linear chromosome and linear plasmid structures were validated through multiple lines of evidence. The chromosome ends were identified by the presence of near-identical terminal inverted repeats (TIRs) of approximately 9.45 kb (99.61% nucleotide identity over 100% coverage), a hallmark of *Streptomyces* linear replicons [23]. Additionally, mapping of short reads to the assembly endpoints confirmed double-coverage patterns consistent with linear replicon termini [24]. The presence of genes encoding terminal proteins (Tpg) was verified: TpgA2 homologs were identified at approximately 30 kb from the chromosome end and at the terminus of the linear plasmid. The plasmid nature of the second genetic sequence was further supported by the absence of this region in all other sequenced *S. anulatus* genomes included in the comparative analysis (*n* = 40), by its size (233 kb, consistent with *Streptomyces* linear plasmids), and by the enrichment of transposase genes, CRISPR-Cas components, and defence-related functions [25, 26].

The combination of MicroScope platform v3.15.3 [27] and Blast2GO 6.0.3 [28] with the Functional Analysis Module of OmicsBox 2.0.36 were employed to annotate the genome and to explore the gene functions. A total of 9,556 CDS were predicted. COG and eggNOG classification assigned 62.47% and 73.18% CDS, respectively. The KEGG metabolic pathways were classified into 11 groups, including amino acid metabolism, carbohydrate metabolism, energy metabolism, and secondary metabolite biosynthesis (Data file 1).

A whole-genome phylogenetic analysis, among approximately 60 publicly available streptomycete genomes, identified DEF39 strain as a member of *Streptomyces anulatus* group. The phylogenetic analysis was performed by submitting the genome to the TYGS server [29], which includes comparisons with the type strain genomes. To improve the *S. anulatus* characterization, this dataset was supplemented with approximately 40 additional *S. anulatus* genomes prioritizing the most complete and high-quality assemblies available in NCBI at the time of analysis. The complete list of genomes included in the taxonomic characterization is provided in Data file 2. The phylogenetic tree was represented using iTOL server (Table 1, Data file 2). Species assignment was further confirmed by Average Nucleotide Identity (ANI) of 99.13% and digital DNA–DNA hybridization (dDDH) of 92.8% with the reference genome GCA_001434355.1.

The antiSMASH web server [30], using strict detection, similarity score of > 0.50 and all extra features enabled, predicted fifteen smBGCs, including non-ribosomal peptide synthetases (NRPS), polyketide synthases (PKS), NRPS–PKS hybrid systems, terpenes, siderophores, lanthipeptides, melanin-related pathways, and ectoine biosynthesis clusters, some of which are associated with abiotic stress protection, bacterial fitness, antibiotics biosynthesis and iron acquisition (Table 1, Data file 3).

PLaBAse v.1.02 platform [31] predicted 5,203 genes putatively associated with plant growth promotion (minimum coverage of 70%; identity of 35%) (Table 1, Data file 4). DEF39 plant growth-promoting traits are potentially associated with plant system colonization (25%), biotic and abiotic stress control (23%), competition for the ecological niche (20%), biofertilization (11%), bioremediation (10%), phytohormone-plant signal production (10%) and plant immune response stimulation (1%). Interestingly, the linear plasmid is enriched for transposons and defence-related genes including a CRISPR-Cas system, endonucleases and genes involved in viral and symbiont response. This enrichment was assessed by comparing the functional annotation of the linear plasmid against the entire DEF39 genome using Blast2GO’s enrichment analysis module. Gene Ontology (GO) terms were considered significantly enriched when the False Discovery Rate (FDR) was below 0.05 (Benjamini-Hochberg correction). Significant enriched GO terms included: transposase activity (FDR < 5.37e-05), response to virus (FDR 0.0495), defense response to symbiont (FDR 0.0495), and DNA metabolic process (FDR 0.0153). Additionally, PLaBAse annotations were used to quantify putative plant growth-promoting traits (PGPTs) encoded on the plasmid relative to the whole genome, based on the PGPT ontology classification. Most adaptive functions (86% of plant growth-promoting traits) are linked to competitive exclusion and antimicrobial resistance. No canonical conjugation genes or secondary metabolite biosynthetic gene clusters were predicted on the plasmid. Overall, these findings suggest a role for DEF39 plasmid in genome plasticity and strain fitness rather than primary metabolism or secondary metabolite production.

**Table 1:** Overview of data files/data sets

Label	Name of data file/data set	File types (file extension)	Data repository and identifier (DOI or accession number)
Data file 1	Genomic features	Table file (.docx)	Figshare https://doi.org/10.6084/m9.figshare.32012037 [32]
Data file 2	Phylogenetic tree based on whole-genome	Figure file (.pdf)	Figshare https://doi.org/10.6084/m9.figshare.32012037 [32]
Data file 3	antiSMASH output	Table file (.docx)	Figshare https://doi.org/10.6084/m9.figshare.32012037 [32]
Data file 4	PLaBAse output	Table file (.xlsx)	Figshare https://doi.org/10.6084/m9.figshare.32012037 [32]
Data set 1	Raw sequence reads	FASTQ files (.fastq)	European Nucleotide Archive ERR16087732 (long reads) https://identifiers.org/ena.embl: ERR16087732, ERR16087731 (short reads) https://identifiers.org/ena.embl: ERR16087731 [33]
Data set 2	Complete annotated genome sequence	GenBank/Annotation Files	NCBI GCA_978019405.1 https://identifiers.org/ncbi/insdc.gca:GCA_978019405.1 [34]

## Limitations

The integration of transcriptomic and metabolomic data would be useful for a more comprehensive and functional interpretation of genomic findings, confirming the putative predictions of the genome analysis via databases.

Genome-based functional prediction is only indicative of the role that identified secondary metabolites and proteins may play in the effective positive activity as biocontrol agent and PGPB of DEF39. Experimental functional analysis of genes and clusters of DEF39 would allow the confirmation of the role played by the set of genes identified.

The inclusion of all the *S. anulatus* genomes available in the NCBI database would provide an even more complete assessment of diversity within *S. anulatus* clade. 

## Data Availability

The datasets supporting this article are available on Figshare at the following link: https://doi.org/10.6084/m9.figshare.32012037. Raw sequencing data (SRA) and the genome assembly (GCA_978019405.1) have been deposited in the European Nucleotide Archive (ENA) under the project accession PRJEB105591.

## Abbreviations

bp: Base pair
GC: Guanine + cytosine content
CDS: Coding DNA sequences
antiSMASH: Antibiotics and secondary metabolite analysis shell
PLaBAse: PLant-associated bacteria server
PGPB: Plant growth promoting bacteria
PGPTs: Plant growth promoting traits
BGC: Biosynthetic gene clusters
smBGC: Secondary metabolite biosynthetic gene clusters
FHB: Fusarium head blight
DON: Deoxynivalenol
ONT: Oxford nanopore technology
COG: Clusters of orthologous groups
eggNOG: Evolutionary genealogy of genes: Non-supervised orthologous groups
KEGG: Kyoto encyclopedia of genes and genomes
ANI: Average nucleotide identity
NCBI: National centre for biotechnology
dDDH: Digital DNA–DNA hybridization
NRPS: Non-ribosomal peptide synthetases
PKS: Polyketide synthases
